# The impact of a cancer Survivorship Care Plan on gynecological cancer patient and health care provider reported outcomes (ROGY Care): study protocol for a pragmatic cluster randomized controlled trial

**DOI:** 10.1186/1745-6215-12-256

**Published:** 2011-12-05

**Authors:** Lonneke V van de Poll-Franse, Kim AH Nicolaije, Maria C Vos, Johanna MA Pijnenborg, Dorry Boll, Olga Husson, Nicole PM Ezendam, Erik A Boss, Ralph HM Hermans, Karin CM Engelhart, Joke E Haartsen, Brenda M Pijlman, Harrie WH Feijen, Helena JMM Mertens, Willem E Nolting, Johannes J van Beek, Jan A Roukema, Roy FPM Kruitwagen

**Affiliations:** 1 CoRPS - Center of Research on Psychology in Somatic diseases, Department of Medical Psychology, Tilburg University, Tilburg, The Netherlands; 2 Eindhoven Cancer Registry, Comprehensive Cancer Center South (CCCS), Eindhoven, The Netherlands; 3 Department of Gynecology, St. Elisabeth Hospital, Tilburg, The Netherlands; 4 Department of Gynecology, TweeSteden Hospital, Tilburg and Waalwijk, The Netherlands; 5 Department of Gynecology, Maxima Medical Center, Veldhoven and Eindhoven, The Netherlands; 6 Department of Gynecology, Catharina Hospital, Eindhoven, The Netherlands; 7 Department of Gynecology, St. Anna Hospital, Geldrop and Eindhoven, The Netherlands; 8 Department of Gynecology, Elkerliek Hospital, Helmond, The Netherlands; 9 Department of Gynecology, Jeroen Bosch Hospital, 's-Hertogenbosch, The Netherlands; 10 Department of Gynecology, Amphia Hospital, Breda and Oosterhout, The Netherlands; 11 Department of Gynecology, Orbis Medical Center, Sittard, The Netherlands; 12 Department of Gynecology, St. Jans Hospital, Weert, The Netherlands; 13 Department of Gynecology, VieCuri Hospital, Venlo and Venray, The Netherlands; 14 Department of Surgery, St. Elisabeth Hospital, Tilburg, The Netherlands; 15 Department of Gynecology and GROW - School for Oncology and Developmental Biology, Maastricht University Medical Center, Maastricht, The Netherlands

**Keywords:** Survivorship Care Plan, Pragmatic cluster randomized controlled trial, Gynecological cancer survivors, Endometrial cancer, Ovarian cancer, Health care providers, Patient Reported Outcomes, Satisfaction with information provision, Satisfaction with care, Health- Related Quality of Life

## Abstract

**Background:**

There is a need for improvement of information provision and post-treatment care for cancer survivors. A Survivorship Care Plan (SCP) is recommended by the American Institute of Medicine and the Dutch Health Council, which is a summary of patients' course of treatment as a formal document, and includes recommendations for subsequent cancer surveillance, management of late effects, and strategies for health promotion. Until now, evidence on the effects of implementing the SCP in clinical practice is lacking. The rationale and study design of a pragmatic cluster randomized trial, aiming to assess the impact of SCP care in routine clinical practice, is presented.

**Methods/Design:**

A web-based patient registration system 'Registrationsystem Oncological GYnecology' (ROGY) is used by gynecologists in the South of the Netherlands since 2006. A personalized SCP can automatically be generated out of ROGY. In this pragmatic cluster randomized controlled trial, 12 hospitals are randomized to either 'usual care' or 'SCP care'. In patients with 'usual care', the gynecologist provides care as usual. In patients with 'SCP care', information about the tumor stage and treatment is personally discussed with the patient and a document is handed to the patient. Prospectively, all patients diagnosed with endometrial or ovarian cancer in the participating hospitals will be approached for study participation. Patients will complete questionnaires after surgery, and before additional treatment, and after 6, 12, 18 and 24 months. In addition, health care providers will be asked their opinion about implementation of SCP care. Primary outcome is defined as patient satisfaction with information provision and care. Secondary outcomes are illness perception, health-related quality of life, health care use, prevalence, course and referral rate of survivors with psychosocial distress, and health care providers' evaluation of SCP care.

**Discussion:**

The ROGY Care trial will help to gain insight into the impact of SCP care on patient reported outcomes, and on the evaluation of cancer survivors and health care providers of the different elements of the SCP. Therefore, results will contribute to efforts to improve quality of care for cancer survivors.

**Trial registration:**

Trial Registration: http://www.ClinicalTrials.gov. Identifier: NCT01185626

Medical Research Ethics Committee Reference Number: NL33429.008.10 Grant Reference Number: UVT2010-4743

## Background

Due to earlier diagnoses and improved treatments, the number of cancer survivors is rapidly increasing in the Western world. In 2000, there were about 400,000 cancer survivors in The Netherlands, and this number is expected to increase to 700,000 in 2015 [1]. Nevertheless, these cancer survivors remain at risk for adverse long-term or late effects of cancer diagnosis and treatment in many physical and psychosocial domains. Quality of life and patient reported outcomes are increasingly acknowledged to be important indicators of treatment efficacy [2], especially since many new therapies only marginally improve survival rates. Furthermore, little is known about the quality of life, psychosocial well-being, and health care needs of the increasing cohort of long-term cancer survivors [3]. There is a need for optimizing the transition from cancer patient to cancer survivor by improving the coordination of post-treatment care for cancer survivors. In the report 'Follow-up in oncology' [4], the Dutch Health Council states that the main goal of aftercare is to provide information and identify and treat the long-term effects of cancer or its therapy. Although psychosocial care is considered to be of major importance, the optimum form during aftercare remains uncertain. According to the Dutch Health Council it is necessary to encourage and give priority to research in this area.

A key factor in the support for cancer survivors is information provision. The information given to cancer survivors about their type of cancer, treatment, possible long-term and late effects and referral services can influence their illness perception and quality of life. It can result in better informed decision making, lower levels of depression and anxiety [5], improved satisfaction with care, sense of control, and quality of life [6]. However, the number of studies that have investigated these associations is limited [7]. Health care providers are often still reluctant to give a full amount of information about prognoses and negative side effects of cancer and its treatment [8]. Although the number of studies that include long-term cancer survivors and their information needs are scarce, results from a few very recent studies suggest that most cancer survivors want more information than is provided by specialists [5,9-11]. These studies underline the importance of a patient-tailored approach to information provision. Providing information that is congruent with a patient's needs at that particular time is an important determinant for patient satisfaction and affects health-related quality of life (HRQoL) and anxiety and depression levels of cancer survivors [12].

An approach to aftercare for cancer survivors recommended by both the American Institute of Medicine (IOM) and Health Council of the Netherlands is the Survivorship Care Plan (SCP). An SCP provides cancer survivors completing primary care with a summary of their course of treatment as a formal document that also includes recommendations for subsequent cancer surveillance, management of late effects, and strategies for health promotion. Essential in such an SCP is detailed information provision about diagnosis and treatment of cancer, possible long-term and late effects, life-style recommendations, and available resources [13]. Based on its 'face validity', the IOM recommends that SCPs become standard of care, as they are likely to improve care [13]. The Dutch Health Council advises the implementation of SCPs for each cancer survivor in the Netherlands. The SCP is expected to be an empowering and enabling device, by facilitating better understanding and self-care by the patient. However, evidence concerning the positive and negative effects of the implementation of the SCP in daily clinical practice is lacking. As such, both the IOM and Health Council also recommend studies for the evaluation of the impact of SCPs on patient and health care provider reported outcomes. Literature suggests a patient-tailored approach to be optimal when providing prognosis or treatment summaries [12]. Health communication strategies, such as SCPs, may have excellent potential to meet the information needs of the increasing group of cancer survivors, and are likely to improve their quality of life [9]. Nevertheless, the psychological impact of these strategies on patient reported outcomes such as perceived quality of information provision, quality of care, quality of life and health care use remains unknown. As such, prospective evaluations of these strategies need to be conducted [9]. Therefore, it is necessary to assess the impact of SCP care in routine clinical practice before its large-scale implementation.

## Methods/Design

### Objectives and hypotheses

The aim of the proposed study is to assess the impact of SCP care on patient and health care provider reported outcomes in routine clinical practice. Primary outcomes include patients' satisfaction with information provision, and satisfaction with care. Secondary outcomes include patients' health-related quality of life, illness perception, health care use, prevalence, course and referral rate of patients with psychosocial distress, and health care providers' evaluation of the (implementation of the) SCP.

It is hypothesized that patients who receive SCP care report better satisfaction with information provision, better satisfaction with care, and more adequate illness perception than those who receive usual care. It is furthermore hypothesized that patients who receive SCP care will report a higher health care use compared to those receiving usual care [14]. In addition, it is expected that patients who receive the SCP will report less anxiety, less depression or psychosocial stress and better HRQoL. However, there may be subgroups (dependent on patient characteristics, such as age, education, and personality) that will be influenced in a negative way when receiving more information than they can handle. Finally, it is hypothesized that patients who receive SCP care are more adequately referred to other health care services when they have high distress levels compared to those who receive usual care.

### Design

A pragmatic, cluster randomized controlled clinical trial (RCT) will be conducted, in which 12 hospitals will be randomized to either 'usual care' or 'SCP care'. It will be a longitudinal study, including patients immediately after initial surgery and following them for 24 months. The trial has been registered on http://www.ClinicalTrials.gov (NCT01185626). The description of this design follows the CONSORT recommendations for reporting on trials (http://www.consort-statement.org) with the extensions for pragmatic [15] and cluster [16] randomized trials.

### Study population

The RCT will be performed in a setting with gynecological patients in 12 hospitals in the South of the Netherlands. These 12 hospitals, including teaching and non-teaching hospitals, will be randomized to either 'usual care' or 'SCP care'. As defined by the US National Coalition for Cancer Survivorship (NCCS), a cancer survivor is: 'A person diagnosed with cancer from the moment of diagnosis through the balance of his or her life' (http://www.canceradvocacy.org). Following this definition, all endometrial and ovarian cancer survivors from the 12 participating hospitals will be included immediately after initial diagnosis and followed for 24 months. Survivors with advanced cancer or those who develop a recurrence or metastasis will not be excluded, since they are all considered to be cancer survivors according to the NCCS definition.

### Inclusion criteria

a) Age ≥ 18 years (no upper age limit)

b) Diagnosed with endometrial or ovarian cancer

### Exclusion criteria

a) Patients with borderline ovarian cancer

b) Patients undergoing palliative care

c) Patients who are not able to complete a Dutch questionnaire

### Recruitment

Patients will be invited to participate in the study by their own gynecologist, after initial diagnosis. The gynecologists will send all their patients the first questionnaire, together with a letter and leaflet to inform them about the study and an informed consent form. The letter and accompanying leaflet about the study will be rather generic, stating the study's objective to investigate the quality of life of cancer survivors. Patients can fill in the informed consent form and complete the questionnaire by pencil and paper and send it back to the researchers in a pre-stamped envelope. If they prefer online completion, they can complete the questionnaire via the PROFILES (Patient Reported Outcomes Following Initial treatment and Long term Evaluation of Survivorship) website http://www.profielstudie.nl[17] after secured login, for which they are provided with a link and password. To guarantee anonymity, returned questionnaires contain only a study number. If the questionnaire is not returned within 1 month, a reminder will be sent, again by the treating gynecologist. After the initial contact via the gynecologist and obtaining informed consent, the follow-up questionnaires at 6, 12, 18 and 24 months after diagnosis will be sent directly to the home address of the patient.

### Randomization

Hospitals have been pre-randomized whereby patients from a certain hospital will receive either usual care and information provision or an SCP. With this so-called pre-randomization, the conventional sequence of obtaining informed consent followed by randomization is reversed [18]. Pre-randomization is justified if valid evaluation of the effects of an intervention is impossible using a conventional randomized design, for example if knowledge of the intervention may lead to non-compliance or drop-out in the control group, or when the intervention is an educational program [18]. Pre-randomization at hospital level eliminates the problem posed by randomization at patient level in which health care providers from a hospital have to provide both types of care. By having to switch between usual care and SCP care for each patient, the health care provider could (unconsciously) influence the usual care group with that of the SCP approach by giving a patient randomized to usual care more information than was intended for usual care. Also, if usual care patients learn from other patients that they are given an SCP, it is possible that they will become dissatisfied with their usual care, even if the (dis)advantages are not well studied. This would bias the results of the study. To prevent imbalance between the treatment groups, stratified randomization was performed according to whether a hospital has a Gynecologic Oncology Center, and the annual number of endometrial and ovarian cancer patients, allowing for an even distribution of the intervention through the different participating centers.

Randomization to either usual care or SCP care at hospital level was performed with a table of random numbers by a researcher not involved in the study and blind to the identity of the hospitals. Because the health care providers administering the intervention have to know whether they have to provide either usual care or SCP care, it was not possible for them to be blinded to the group assignment. The participants on the other hand are unaware of the group assignment, as they are under the assumption that the hospital is providing usual care.

### Intervention versus usual care

*Usual care*: The health care provider (i.e., gynecologist or oncology nurse) provides care as usual. Currently, the 12 involved hospitals provide follow-up following the Dutch guidelines http://www.oncoline.nl, meaning that they see their patients on given time points based on the number of years after diagnosis. Most of the participating hospitals give their patients leaflets regarding the diagnosis and treatment they receive. However none of them provide personalized printed information. All information is given during the initial treatment phase, but none of the health care providers give additional information during follow-up, except for ad-hoc referrals if needed by the patient. Most of the health care providers are not actively screening on psychosocial needs. As the usual care and information provision in these hospitals might change in time, the health care providers and patients will be asked about the type of information (e.g., brochures, DVDs, websites, personalized information) and psychosocial care they provide and receive, respectively.

*SCP care*: The health care provider (i.e., gynecologist or oncology nurse) provides the patient with a paper SCP after initial treatment, and discusses all the items in the SCP with the patient. To improve communication, the health care provider also sends a copy of the SCP to the patient's general practitioner. In follow-up consultations, the patient will receive an updated SCP if applicable. The health care providers involved in the SCP arm attended an instruction-evening to enhance the complete use of the SCP, by providing them with practical guidelines on how to discuss the SCP information with patients. In addition, the health care providers received a reminder, consisting of a summary of the purpose of the SCP and the study, and practical guidelines of the use of the SCP, intended to remind the health care providers to provide an SCP to their patients. As this is a pragmatic trial aiming to assess the impact of SCP care in daily clinical practice, the delivery of the intervention is allowed to vary between health care providers. The health care providers have the flexibility to discuss the SCP according to the patients' needs.

### ROGY Survivorship Care Plan

For the development of the SCP used in this RCT, the Dutch SCP template, which is very similar to the format that was described by the IOM [13], was adjusted to the local situation as was suggested by Ganz [19]. A subgroup of gynecologists, oncology nurses, a radiotherapist, a medical oncologist, as well as a general practitioner and a few patients adjusted a standardized SCP to the local situation.

The paper SCP is extracted from the online registration system 'ROGY' (Registrationsystem Oncological GYnecology) and provides tailored information based on personal patient and disease data (e.g., name patient, date of birth, type of cancer, cancer stage, treatment received, providers involved). Detailed information is provided on the diagnosis, treatment, possible short-term and long-term effects of the disease and the treatment, and aftercare. In addition, the Distress Thermometer [20] is provided as an aid for the communication about psychosocial distress between patients and health care providers.

The SCP contains information that is tailored to the specific situation of the patient. For instance, a patient who received adjuvant radiotherapy will get information about potential long-term effects of radiotherapy and what to do if certain complaints arise. Recurrences, toxicities or other specialists involved in the patient's care will be registered in ROGY and automatically updated in the personal SCP.

The ROGY system was set up to facilitate patient registration and improve patient care by means of uniform regional multidisciplinary patient consultation. For each patient, a detailed registration is made, including information about FIGO (International Federation of Gynecology and Obstetrics) stage, grade, treatment, complications, comorbidity and follow-up. Thus, all necessary information for an SCP [12] is already routinely registered by all participating gynecologists, in a uniform way. By pressing a button in ROGY, which is only visible for the health care providers randomized to SCP care, it is possible to automatically generate a personalized SCP from the information available in the ROGY system. All gynecologists randomized to either usual care or SCP care use this registration system, so there will be no registration bias. Therefore, the possible quality of care improvement that comes with detailed registration is equal in both arms, and only the impact of providing SCPs will be evaluated.

### Patient reported outcomes

Outcomes will be assessed with standardized and validated measures shown to have acceptable psychometric properties.

*Satisfaction with information provision *will be measured using the EORTC-INFO26 [21] module. This questionnaire aims to evaluate the (satisfaction with) information received by cancer patients on different areas of the disease, diagnosis, treatment and care, and some qualitative aspects, for instance wishes for more information. The questionnaire contains the following scales: (a) Information about the disease; (b) Information about medical tests; (c) Information about treatment; (d) Information on other services, and single items: (a) Written information; (b) Information on CDs or tape/video; (c) Satisfaction with the amount of information; (d) Desire for more information; (e) Desire for less information; (f) Helpfulness of information. Furthermore, a more specific question will be added about whether patients have received an SCP, to control for physician compliance with the provision of SCP care.

*Cancer specific HRQoL *will be measured with the EORTC QLQ-C30 [22]. Much of the content of the questionnaire is appropriate for extended monitoring of health status, including scales assessing physical, cognitive and emotional functioning, fatigue and sleep problems, and overall health and quality of life. This core instrument is supplemented by a condition-specific questionnaire module. For endometrial cancer, the EORTC-EN24 [23] module will be used. This module assesses lymphoedema, urological symptoms, gastrointestinal symptoms, body image, sexual/vaginal symptoms, back/pelvic pain, and chemotherapy side effects. For ovarian cancer, the EORTC-OV28 module [24] will be used, which measures abdominal/gastrointestinal symptoms, peripheral neuropathy, other chemotherapy side-effects, hormonal/menopausal symptoms, body image, attitude to disease/treatment and sexual functioning.

*Satisfaction with care *will be measured with the EORTC IN-PATSAT32 [25]. This questionnaire was designed to assess cancer patients' perception of the quality of medical care, nursing care and care organization and services received in the hospital. The EORTC IN-PATSAT32 contains 11 multi-item and 3 single-item scales. These include the doctors' technical skills, interpersonal skills, information provision, and availability; the nurses' technical skills, interpersonal skills, information provision, and availability; the other hospital staff's interpersonal skills and information provision scale; the exchange of information single-item scale; the waiting time; the hospital access; the comfort single-item scale; and the general satisfaction single-item scale.

*Illness perception *will be measured using the Brief Illness Perception Questionnaire (B-IPQ) [26]. The scale has 9 items, measuring (a) cognitive representations (consequences, timeline, personal control, treatment control, identity); (b) emotional representations (concern, emotion); (c) illness comprehensibility; and (d) an open-ended response item on the 3 most important causal factors of illness.

*Comorbidity at the time of questionnaire completion *will be assessed by the adapted Self-administered Comorbidity Questionnaire (SCQ) [27]. Patients will be asked to identify comorbid conditions developed in the past 12 months. The adapted SCQ lists 14 medical conditions (with the option to list up to 3 additional conditions), and assesses if the patient is treated for the comorbid condition and the perceived impact on the patients' functioning.

*Anxiety and depression *will be assessed using the Hospital Anxiety and Depression Scale (HADS) [28]. The HADS has 14-items, with 7 items each for anxiety and depression. Each item is scored from 0 to 3, where a score of 3 represents a state corresponding to the worst anxiety or depression. The sum of these items produces two subscales ranging from 0 to 21. A score of 8 will be used as a cut-off value for both depression and anxiety [28].

*Physical and mental fatigue *will be measured using the Fatigue Assessment Scale (FAS) [29], which consists of 10-items with 5 items each reflecting physical and mental fatigue. The items are scored on a 5-point response scale ranging from 1 (never) to 5 (always).

*Personality *will be measured with the DS14 [30] which has 14 items ranged on a 5-point response scale ranging from 0 (false) to 4 (true). Seven of these items refer to 'Negative Affectivity' or the tendency to experience negative emotions in general (e.g., 'I am often down in the dumps or I often find myself worrying about something'). The remaining 7 items refer to the patient's level of 'Social Inhibition', or the tendency to inhibit the expression of emotion/behavior in social relationships (e.g., 'I am a closed kind of person or I often feel inhibited in social interactions'). According to previously published cut-off scores, patients were diagnosed as Type D if they scored ≥10 on both the Negative Affectivity and Social Inhibition scales.

*Health care utilization *will be assessed by patients' frequency and reason (cancer-related or not) for contact with their general practitioner or medical specialist in the past 12 months. Patients will be asked whether they have been referred to other health care services.

Additional measures include demographic and socioeconomic variables such as age, education, marital status, and employment status, and clinical variables such as cancer stage at diagnosis, time after diagnosis, and initial treatment. All measures will be collected at the beginning of the trial, and at 6, 12, 18 and 24 months after diagnosis.

### Health care providers' evaluation

*Gynecologists and oncology nurses *in both arms will be asked their opinion about the implementation of SCP care or usual care. At the beginning of the trial and after 12 months, gynecologists and oncology nurses will be asked to fill in a short questionnaire containing questions about the type of information they provide, how satisfied they are with the information they provide, how much time they spend on their consultations on average, to what extent they expect the SCP care to have a positive effect on patients, and how motivated they are to use the SCP. In addition, at the end of patient inclusion after 12 months, all gynecologists and oncology nurses in the SCP arm are invited to participate in qualitative interviews on their opinion about (the implementation of) the SCP care.

*General practitioners *of patients in both arms will be asked to fill in a short questionnaire, containing questions about whether they received the SCP and whether they believe it has improved the communication between themselves and the gynecologist, and between themselves and their patient.

### Sample size

The initial patient inclusion will be completed in approximately one year. Each year, approximately 250 endometrial and 200 ovarian cancer survivors are diagnosed in the participating hospitals. Based on previous studies [31-34] 337 patients (75%) are minimally expected to participate in one year. Allowing for 10% attrition, this equates about 300 patients. Assuming an average practice size, the power will be 0.98 to detect a minimum effect size of 0.5, which is considered clinically significant [35]. In addition, separate analyses for endometrial and ovarian cancer survivors can be conducted: 150 patients are sufficient to detect a half standard deviation difference with a power of 0.83.

### Statistical analysis

Analyses will be performed adhering to the intention-to-treat principle. Descriptive statistics will be used to summarize characteristics of both hospitals and patients. Characteristics of patients (i.e., age, type of cancer, stage, treatment, socio-economic status, marital status, educational level, employment status) and hospitals (i.e., number of endometrial and ovarian cancer patients, whether a hospital is a training hospital) will be compared at baseline between the intervention and usual care group, by regression analyses.

Linear regression models will be used to analyze continuous outcome variables, and logistic models for binary outcome variables. All analyses will be adjusted for hospital, baseline values of the outcome measure, and baseline variables which differ to a clinically significant extent between groups. Differential effects of SCP care by age, type of cancer and baseline levels of the outcomes of interest will be assessed for the outcome measures by adding terms for the interaction between age, educational level, personality, type of cancer and baseline levels and care arm to the regression models.

A multilevel modeling approach [36] will be applied to evaluate differences between the intervention group and the control group. This approach will be used to take into account the clustering at the hospital level [37]. All statistical tests will be two-sided and considered significant if *p*<0.05. Repeated measures analysis using generalized estimating equations will be conducted to account for the intra-patient dependency of the repeated measures [38]. Differences in effect of the SCP care and usual care at the different time points will also be investigated [38].

Missing outcome data will be assumed to be 'missing at random' (MAR), conditional on key predictors of 'missingness' (in particular baseline values of the outcome variables of interest, and care arm). Clinically meaningful differences will be determined with Norman's 'rule of thumb', whereby a difference of ≈0.5 *SD *indicates a threshold of discriminant change in health status scores of a chronic illness [35].

Routinely collected data from the population-based Eindhoven Cancer Registry (ECR) and ROGY on patient and tumor characteristics will allow for the comparison of respondents, non-respondents and patients with unverifiable addresses, using t-tests for continuous variables and Chi-square analyses for categorical variables.

### Ethical considerations

The study protocol has been approved by the medical research ethics committees of the participating centers (Medical Research Ethics Committee reference number: NL33429.008.10). The study will be conducted according to the Declaration of Helsinki, as amended in 2008 by the World Medical Association, and all patients will be informed about the purpose, rights, and possible benefits/risks of the study. Study participation of patients is voluntary and can be cancelled at any time without provision of reasons and without any consequences. Patients can call a psychologist, an epidemiologist, or an independent general practitioner for more information about the study.

### Data security/disclosure of original documents

Confidentiality and anonymity of participants will be guaranteed with the assignation of a study number to each participant. As such, returned questionnaires have no names attached and will be linked to data from the ECR by study number. Therefore, it will not be possible for the researchers to track participants' names with the study numbers. The results of the patient questionnaires are not accessible to the gynecologists or other health care providers. Questionnaires are directly mailed to the study center by the patients. Returned questionnaires will be stored in a secured location for five years. Only direct members of the internal study team can access the respective files.

## Discussion

The number cancer survivors that are confronted with adverse long-term or late effects is rapidly increasing. These cancer survivors remain at risk for adverse long-term or late effects of cancer diagnosis and treatment, and often do not know how to interpret their physical or psychosocial problems, or where to go for support. Providing patients with an SCP may be an empowering and enabling instrument, by facilitating better understanding and self-care by the patient. However, evidence concerning the effects of the implementation of the SCP in daily clinical practice is lacking. It is therefore necessary to assess the impact of SCP care in routine clinical practice before its large-scale implementation.

If this study shows that SCP care is feasible and effective, this scientific evidence can be used to convince cancer survivors' health care providers and health insurance companies about the benefits of implementing SCP care. The results will help to gain insight into the impact of SCP care on patient reported outcomes, and on the evaluation of cancer survivors and health care providers of the different elements of the SCP. Therefore, results will contribute to efforts to improve quality of care for cancer survivors.

## Trial status

The inclusion of patients started April 2011. The initial patient inclusion will be completed in approximately one year. Patients will be followed for two years.

## List of abbreviations

CCCS: Comprehensive Cancer Center South; ECR: Eindhoven Cancer Registry; HRQoL: Health-Related Quality of Life; IOM: American Institute of Medicine; MAR: Missing At Random; NCCS: National Coalition for Cancer Survivorship; OOG-CCCS: Organization Oncologic Gynecology of the Comprehensive Cancer Center South; RCT: Randomized Controlled Trial; ROGY: Registrationsystem Oncological GYnecology; SCP: Survivorship Care Plan.

## Competing interests

The authors declare that they have no competing interests.

## Authors' contributions

In collaboration with the OOG-CCCS (Organization Oncologic Gynecology of the Comprehensive Cancer Center South), LP, CV, DB, JP, OH, AR, and RK contributed to the design of the study and provided the basis for the intervention. All authors contributed to the development of the trial protocol and data collection. LP and KN drafted the manuscript. All authors provided input into revisions of the manuscript and have approved the final manuscript.

## Links

ClinicalTrials.gov http://www.ClinicalTrials.gov

National Coalition for Cancer Survivorship http://www.canceradvocacy.org

CONSORT recommendations for reporting on trials http://www.consort-statement.org

PROFILES http://www.profielstudie.nl

Oncoline - Cancer Clinical Practice Guidelines http://www.oncoline.nl
